# Optimizing expectations to prevent side effects and enhance quality of life in breast cancer patients undergoing endocrine therapy: study protocol of a randomized controlled trial

**DOI:** 10.1186/1471-2407-13-426

**Published:** 2013-09-18

**Authors:** Pia von Blanckenburg, Franziska Schuricht, Ute-Susann Albert, Winfried Rief, Yvonne Nestoriuc

**Affiliations:** 1 Department of Clinical Psychology and Psychotherapy, Philipps University, 35032, Marburg, Germany; 2 Department of Gynecology, Gynecological Endocrinology and Oncology, Breast Cancer Center, Philipps University, Marburg, Germany; 3 Department of Clinical Psychology, Hamburg University, Hamburg, Germany

**Keywords:** Nocebo, Side effects, Prevention, Breast cancer, Endocrine therapy, RCT

## Abstract

**Background:**

Adjuvant endocrine therapy can improve disease**-**free survival and time before recurrence in breast cancer patients. However, it is associated with considerable side effects that negatively affect patients’ quality of life and cause non**-**adherence. The recently demonstrated effect of individual expectations on side**-**effect development (nocebo effect) suggests that psychological factors play a role in the prevention of side effects. The aim of this study is to evaluate cognitive**-**behavioral side**-**effect prevention training (SEPT) for breast cancer patients. This article describes the study protocol and applied research methods.

**Methods/Design:**

In a randomized controlled trial, 184 female breast cancer patients are assigned to receive either SEPT, standard medical care or a manualized supportive therapy at the start of adjuvant endocrine treatment. SEPT consists of three sessions of cognitive**-**behavioral training including psychoeducation to provide a realistic view of endocrine therapy, imagination**-**training to integrate positive aspects of medication into daily life, and side**-**effect management to enhance expectations about coping ability. Side effects three months after the start of endocrine therapy serve as primary outcomes. Secondary outcomes include quality of life, coping ability and patients’ medication adherence. Patients’ expectations (i.e., expectations about side effects, coping ability, treatment and illness) are analyzed as mediators.

**Discussion:**

The optimization of expectations might be a potential pathway in health care to improve patients’ quality of life during long**-**term medication intake. The results will provide implications for a possible integration of evidence**-**based prevention training into clinical practice.

**Trial registration:**

ClinicalTrials.gov, (NCT01741883).

## Background

Endocrine therapy is the pivotal adjuvant treatment for over 75% of breast cancer patients. National and international guidelines recommend it for long**-**term intake, over at least five years, in patients with hormone**-**receptor**-**positive primary breast cancer [1,2]. Adjuvant endocrine therapy reduces the risk of cancer recurrence, development of metastases, and cancer mortality [3,4]. Despite these benefits, almost every second patient discontinues treatment [5], while another 17% refuse to initiate drug intake [6]. Reported non**-**adherence rates range from 35% to 50% [7,8], leading to increased mortality in women with breast cancer [9]. Main reasons for non**-**adherence are side effects that reduce patients’ quality of life [10]. Thus, effective side**-**effect management and side**-**effect prevention are crucial.

Most frequently reported side effects of endocrine therapy are arthralgia, hot flushes, weight gain, mood swings, loss of libido and vaginal dryness [11,12]. To a great extent these symptoms are directly caused by the pharmacodynamics of the treatment, depleting women of female sex hormones, thereby initiating or adding up to typical menopausal discomforts. In contrast, long**-**term evaluations of adjuvant hormonal therapy showed that a substantial proportion of the reported side effects were not related to the treatment [13]. These side effects, such as headaches, skin irritation, dizziness, nausea and gastrointestinal irritation, have no known pharmacological association with endocrine treatment and are therefore termed non**-**specific medication side effects [14]. They are influenced by patient characteristics such as expectations and pre**-**existing symptoms. It is assumed that analog processes are involved in the nocebo (Latin: “I shall harm”) phenomenon, in which placebo**-**pills cause adverse effects [15]. Hence, a randomized placebo**-**controlled study of letrozole showed that around 20% of breast cancer patients in the placebo arm experienced typical menopausal symptoms [16]. Thus, negative expectations can not only influence the occurrence of non**-**specific side effects but even worsen specific side effects via the nocebo**-**mechanism (nocebo effect) [17,18].

Negative expectations about medication have been shown to predict the incidence of non**-**specific side effects in patients with rheumatoid arthritis, even if disease severity and medication regimen were controlled [19]. Expectations about occurrence and intensity of side effects seem to be an important predictor of side effects in cancer treatment [20], for example chemotherapy**-**related nausea [21]. A recent meta**-**analysis showed a significant, medium**-**sized association between patients’ expectations of side effects and the actual experience of these side effects from cancer treatments [22]. In a pilot study by our group, response expectations predicted the incidence of side effects three months after the start of endocrine treatment [23,24]. Furthermore, cognitive representations and expectations about the consequences of illness were found to be associated with physical health outcomes in breast cancer patients [25]. Leventhal’s self**-**regulation model of health [26] additionally focuses on expected coping with illness [27] and the emotional representations of an illness [26]. Thus, anxiety [28] and depression may be relevant in the development of nocebo effects and non**-**specific side effects [14]. Several other factors appear to be of importance in this context: e.g., prior experiences with side effects [22], higher pre**-**existing symptoms [29], and the tendency toward somatization, symptom amplification and selective attention on bodily sensations [14], all of which can result in a possible misinterpretation of prior existing symptoms as side effects of the cancer medication [30]. Taken together, patients’ expectations seem to be essential for the development of side effects. Optimizing these expectations might be a promising way to minimize patients’ side**-**effect burden during long**-**term intake of endocrine medication.

Only few studies have tried to optimize expectations in cancer treatment. Changes in illness representations are associated with less fear of progression [31], and patients with high expectations for treatment**-**induced nausea could profit from a positive expectation**-**manipulation [32]. In particular, the way information about side effects is given to the patients is crucial for the development of expectations. It is recommended that information be framed in a positive way, e.g., not only explain possible side effects but also the expected benefits of the medication. Further, it is important to promote a positive doctor**-**patient interaction and to foster effective management of symptoms [33]. Recent studies showed that cognitive behavioral therapy helps breast cancer patients in the management of menopausal side effects and can lead to decreased levels of reported symptoms [34,35]. So far, no study has focused on optimizing expectations to prevent side effects. We therefore developed a psychological side**-**effect prevention training (SEPT) to prevent side effects by optimizing patients’ expectations, which may be an effective pathway to enhance overall quality of life. This article describes the study design and the research methods to answer the following research questions:

 1. Is a three**-**session psychological intervention effective in reducing side effects and improving quality of life during long**-**term intake of endocrine therapy?

 2. Do treatment and side**-**effect expectations mediate the beneficial effects?

 3. Are there certain patient characteristics that predict which patients benefit the most from the training?

## Methods/Design

### Study design

**Figure 1:**
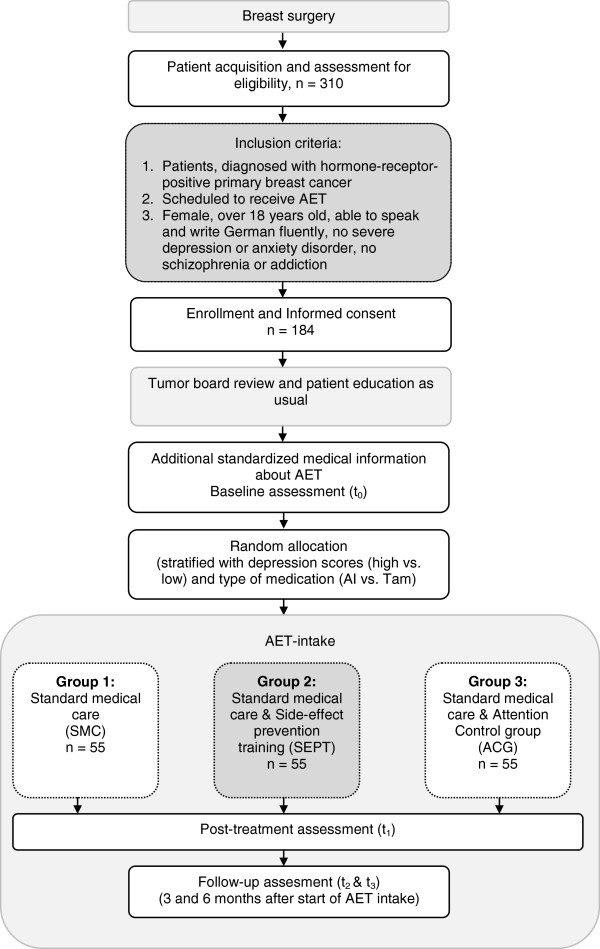
Study design.

The study is designed as dual**-**center, randomized controlled trial with three arms and follow**-**up assessment (see Figure 1). The study procedure is implemented at the Department of Gynecology, Gynecological Endocrinology and Oncology, Philipps University Marburg, Germany, and at the University Medical Center Hamburg**-**Eppendorf, Germany. Participants are women with hormone**-**receptor**-**positive breast cancer, scheduled to start adjuvant endocrine treatment. After signing informed consent to participate in the study and receiving medical information about endocrine treatment from the hospital staff, all patients are provided additional standardized information and patient education about the scheduled treatment by a trained research assistant/ clinical psychologist to homogenize knowledge about endocrine treatment. The structured treatment information is given verbally and through a leaflet, illustrating the physical mode of action, the desired effects, and the potential side effects of endocrine therapy. After completing baseline assessment (including symptom status and pre**-**treatment side**-**effect expectations) patients are randomly assigned to one of three groups. Group 1 receives standard medical care (SMC) only. Group 2 receives SMC and the side**-**effect prevention training (SEPT). Group 3 receives SMC and supportive therapy as an attention control group (ACG). Outcomes are assessed homogeneously in all three groups, three and six months after the start of medication intake.

### Participants

Patients with hormone**-**receptor**-**positive primary breast cancer scheduled to undergo first**-**line endocrine treatment with tamoxifen (and additional GnRH**-**analoga, depending on the menopausal status) or a third**-**generation aromatase inhibitor (i.e., anastrozole, letrozole or exemestan) are eligible. Exclusion criteria are the presence of a serious co**-**morbid psychiatric condition (schizophrenia, addiction, severe affective or severe anxiety disorder), the presence of a life**-**threatening co**-**morbid (non**-**cancer) medical condition, insufficient German language skills, and cognitive inability to give informed consent. Inclusion and exclusion criteria are checked by screening patients’ history and medical records and standardized assessment using the structured psychiatric interview “mini**-**dips” [36].

### Recruitment and informed consent

Diagnosis and treatment are initiated independently of the study according to the German breast cancer guidelines by the interdisciplinary tumor board of the breast cancer center. Patients are required to have completed primary surgery and/or chemotherapy, if indicated.

Patients are screened for eligibility during their hospital stay in the breast cancer center. Women meeting the inclusion criteria are informed about the study concept and invited by their oncologists to take part in the study. Patients who give written informed consent after receiving detailed written and verbal information are admitted to the study. Participation is voluntary and can be withdrawn by the patient any time with no disadvantages.

### Ethics

The study protocol was approved by the medical ethics committee of the Philipps University Marburg, and the Hamburg Medical Chamber. The study will be conducted in accordance with the Declaration of Helsinki, Good Clinical Practice guidelines, including data and patients’ privacy protection.

### Sample size calculation

Required sample sizes were determined a priori with G**-**Power [37]: The sample size for the randomized group comparison was determined according to a MANOVA with repeated measures, testing for a three groups x three time points interaction. Assuming a medium effect size of f(V)=.20, N=153 patients (n=51 per group) are needed to achieve 80% power.

Taking into account a potential drop**-**out rate of 20% (e.g., due to exhaustion or medical complications), we aim to include a total of 184 breast cancer patients.

### Quality standards (Minimization of bias)

#### Randomization and blinding

Randomization follows after completing baseline assessment. Patients are equally allocated to one of the three treatment arms using sealed envelopes. Assignment follows a stratified permuted block randomization procedure with a block size of nine. Stratification criteria (2×2) are the sum score (≤13 vs. >13) of the hospital anxiety and depression questionnaire (HADS**-**D) [38,39] during hospital stay and type of medication (aromatase inhibitor vs. tamoxifen). Assignment sequence is generated by staff who are not involved in the intervention process and conducted electronically using the statistical program WINPEPI [40]. The research assistants responsible for the assessment are blinded to group allocation.

#### Attrition bias

In order to examine potential attrition bias, drop**-**out analyses will be performed. In accordance with the intention**-**to**-**treat principle, the data of all patients randomised to the treatment groups will be analysed.

#### Control for therapeutic allegiance

To ensure the comparability of treatments, treatment dose, application and assessment occasions for SEPT and ACG will be identical.

All therapists are clinical psychologists with advanced cognitive behavioral training and have comparable professional experience. They are trained in the use of the treatment manual before the trial starts. Training includes a full treatment cycle with at least one patient per treatment group using video feedback and professional supervision of each treatment session. During the whole study therapists are under ongoing supervision by highly experienced psycho**-**oncologists. Both types of interventions are manualized and each therapist will treat a comparable number of patients in each group. Treatment fidelity is assessed and rated before approval of therapists to start in this trial. All sessions will be videotaped and an amount of 33% will be selected randomly and rated by an independent rater.

### Psychological interventions

**Figure 2:**
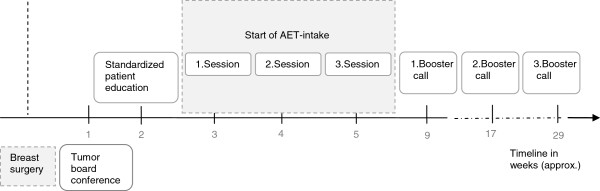
Intervention schedule.

In the intervention groups three individual sessions of 50–75 minutes are being held with a clinical psychologist over the course of three to four weeks (see Figure 2). One, three and six months after the intervention booster telephone calls will be made in both intervention groups.

#### Intervention: side-effect prevention training

The goal of SEPT is the prevention of non**-**specific and nocebo side effects from endocrine therapy by optimizing treatment**-** and illness**-**related expectations. Contents of the training are psychoeducation [41] to provide a realistic view of the treatment, imagination**-**training to integrate positive aspects of medication into daily life and side**-**effect management [42] to enhance expectations about individual coping abilities. All intervention components and goals are listed in Table 1. SEPT is a manual**-**based program. Individual topics are adapted specifically to each patient according to her individual expectations. The baseline questionnaires are used as a starting point for tailoring the intervention so it is consistent with the written treatment guidelines. Patients in the SEPT group receive a booklet with patient material detailing the contents, goals and interventions of each session. The booklet also contains work sheets, postcards and further material that can be personalized by each participant. Sessions will focus on subsequent topics:

**Table 1:** Intervention components and goals of the side-effect prevention training

**Intervention components**	**Goals**
**Session 1**	
- Psychoeducation about AET	- Knowledge about AET and nocebo effect
- Guided imagination and visualization of positive treatment aspects	- Strengthen control and benefit expectations
- Psychoeducation about nocebo and non-specific side effects	- Integration of positive aspects of AET into daily routines
**Homework**	
- Practice relaxation and imagination (anchored by CD)	
- Further creative work with imagination, e.g., painting	
**Session 2**	
- Develop individual problem-solving scheme for the three most important side effects	- Optimize coping expectations
- Create an action plan for behavioral and cognitive strategies	- Reduce specific concerns
**Homework**	
- Complete and modify the personal problem-solving scheme	
- Create an individual “tool box”	
- Practice relaxation and imagination (anchored by CD)	
**Session 3**	
- Psychoeducation about doctor-patient communication	- Optimizing coping expectations
- Develop distraction strategies of for the time of AET intake	- Improving patient-physician communication
- Summing up and outline treatment goals	
**Homework**	
- Practice relaxation and imagination (anchored by CD)	
- Complete and modify the personal problem-solving scheme and the tool box	
**Telefone booster calls**	
- Compare expected and occurred side effects	- Maintain optimized expectations
- Check practicability of the coping strategies and the “tool box” and modify if necessary	

Notes: *AET* = Adjuvant endocrine therapy

*Session 1:* At the beginning of the first session, psychoeducation about the active principle of endocrine treatment is given, tailored to the individual patient’s needs. The adverse side effects most expected by the patient are discussed and contrasted with treatment benefits.

A guided imagination is performed to visualize the positive aspects (e.g., protection from cancer recurrence) of the endocrine treatment. The imagination is recorded on audio file and incorporated into patients’ daily handling of the medication. Furthermore, the impact of expectations on side effects (e.g., pain following negative expectations but without the administration of any inert substance) is discussed and the concept “nocebo phenomenon” explained to the patients.

*Session 2:* Session two focuses on the development of coping strategies for side-effect management that are implemented in a written problem**-**solving scheme for the three most expected or dreaded side effects. Strategies employed include behavioral techniques, cognitive strategies, dietary advice, physical exercises, identification and avoidance of triggers for specific side effects. Patients are asked to create a “tool**-**box” at home filled with useful material to implement the discussed coping**-**strategies.

*Session 3:* This last session includes skills training for improved patient**-**physician communication to positively influence expectations about breast cancer check**-**ups. Additionally, the role of attention for the development of non**-**specific side effects and the worsening of specific side effects is discussed. Patients are encouraged to re(activate) individual resources and activities helping to distract from the potential occurence of side effects, but also strenghten the patient for the time of medication intake. At the end of the session, all topics of the previous sessions are reviewed.

*Booster calls:* The three phone calls focus on repeating the contents of the intervention. The therapeutic contact is used to give support to the patients during the first time of medication intake. Many patients describe it as very helpful to talk about their concerns in this phase of illness and treatment (the phase of rehabilitation after being discharged from hospital). During the booster calls, possible problems with the relaxation and imagination training are discussed and resolved whenever possible. The coping strategies are reviewed and adapted. Furthermore, the problem**-**solving scheme will be extended to further side effects including new coping strategies.

### Attention control group (ACG): *supportive therapy*

Supportive therapy serves as an attention control group for non**-**specific factors such as therapist’s attention and patient**-**therapist relationship. It serves to distinguish specific effects of SEPT from psychological placebo effects [43]. Supportive therapy [44] as a manualized, non**-**specific psychological intervention has been previously used in clinical trials [45]. It includes common factors such as elicitation of affect, treatment context, empathy, reflective listening, and feeling understood. It will be delivered in the same frequency and on the same occasions as SEPT (three individual sessions and three booster calls).

*Sessions 1, 2 and 3:* In the supportive therapy session contents may vary; there are no specific topics therapists need to address, there is no patient material and no homework. Nevertheless, every session can be structured into three phases: the beginning, the therapeutic dialog and the end. At the beginning patients are asked about relevant themes they want to talk about. During the therapeutic dialog patients lead the session and the therapist follows, while concentrating especially on the validation of patients’ affects. It is suggested to talk about any topics that appear to have an affective valence to the patients. The therapist focuses on creating a warm atmosphere and shows empathy and unconditional positive regard towards the patient. At the ending of all sessions all themes are reviewed with the focus on the affect of the individual patient.

In the control group booster calls are conducted simultaneously to those in the intervention group, but they do not follow a particular structure. Patients are asked about their feelings since the last session.

### Measures

Assessment occurs at four measurement points (see Table 2): at baseline approximately two weeks after surgery, after the intervention (post**-**intervention), and three and six months after the start of medication intake (follow**-**up). Questionnaires are applied by blinded research assistants at all measurement points. The following demographic and medical information will be obtained from medical charts and by baseline**-**interviews: socio**-**demographic status, age, BMI, health status, accompanying illnesses and medication (e.g., osteoporosis and medication), prior experiences with endocrine treatment (hormone contraception, hormone replacement therapy), stage of disease and tumor characteristics (UICC**-**stage, TNM**-**classification, Grade, ER/PR status, Her**-**2**-**status), type and course of primary treatment (breast**-**conserving surgery, mastectomy, radiotherapy, systemic treatment). Medical follow**-**up data include health and disease status, accompanying illnesses and medication for clinical outcome. All data will be validated and a plausibility check**-**up will be conducted.

**Table 2:** Study measures

		**Baseline pre-intervention**	**Post-intervention**	**Follow-up (3 and 6 months after AET intake)**
Inclusion criteria	**Interview measures**			
	Structured psychiatric interview (mini-dips)	x^1^		
	Demographic and medical data	x		
	**Questionnaire measures**			
Primary + Secondary Outcomes	Physical symptoms and side effects + coping (GASE+Coping)	x		x
	Quality of life (EORTC QLQ C30 & BR23)	x		x
	Adherence (MARS-D), Adherence Intention	x	x	x
Expectation Scales	Expected side effects + expected coping (GASE-Expect + * *Coping)	x	x	x
	Illness beliefs (IPQ-B)	x	x	x
	Beliefs about medicines (BMQ-D)	x	x	x
Process Variables	Fear of progression (PA-F-K)	x	x	x
	Anxiety and depression (HADS)	x	x	x
	Knowledge hormone receptor	x	x	x
	Treatment evaluation		x^2^	
Control Variables	Somatosensory amplification (SSAS)	x		
	Prior experiences with endocrine treatment	x		
	Partnership quality	x		
	Additional medication	x		x

Notes:

^1^The psychiatric interview is conducted prior to the measurement to check the inclusion criteria.

^2^Completed after every therapy session in the intervention groups only.

#### Primary outcomes

*Side effects* will be measured with the General Assessment of Side Effects scale (GASE) [46,47] which systematically assesses incidence and intensity of the 36 most common side effects. To assess the most frequent patient**-**reported adverse side effects from endocrine treatment the scale was modified by adding nine further symptoms (decreased interest in sex, weight gain, feeling of tension in breast, mood swings, abdominal bloating, vaginal dryness, bone fracture, pain during sexual intercourse, cataract) resulting in a total of 45 symptoms. Patients will be asked to rate the intensity of each listed bodily complaint during the past seven days on a four point Likert scale (0 = “complaint not present”; to 3 = “severe intensity”). Additionally, patients will indicate whether they attribute each of the present symptoms to current drug intake. It shows satisfactory psychometric properties [46].

#### Secondary outcomes

*Quality of life* will be assessed with the European Organization for Research and Treatment of Cancer Quality of Life Questionnaire (EORTC QLQ**-**C30) with breast module (QLQ**-**BR23) [48]. This questionnaire can be seen as the standard multidimensional instrument to measure quality of life among cancer patients. The QLQ**-**C30 consists of five functional scales, nine symptom scales and a global quality**-**of**-**life scale. The breast module is composed of eight breast cancer specific scales including four functional and four symptom scales. Both questionnaires have demonstrated good psychometric properties [48,49].

*Coping ability* measures the perceived ability to manage occurring side effects. It will be accessed via a modified version of the GASE [46,47] that will ask patients to rate their coping ability for each of the 45 listed side effects (i.e., “How good is your ability to manage the occurring adverse symptom?) on a Likert scale from 0 = “good” to 4 = “bad”.

*Medication adherence* will be measured with the German version of the Medication Adherence Report Scale (MARS) [50,51]. This scale gives an indication of the extent to which non**-**adherent behaviors occur, including how often patients have consciously not taken their medicines or forgotten to take them. It has been used to measure the adherence in endocrine treatment before [52]. Additionally, the *adherence intention*, the *actual adherence* and the *attitude towards* in cursive will be assessed with three single items.

#### Expectations

*Side effect expectations* refer to the patients’ cognitive representations of the undesired effects related to a specific treatment. They will be measured with the General Assessment of Expected Side Effects Scale (GASE**-**expect), which is a modified version of the GASE**-**scale [46,47] and was designed to measure patients’ pharmacological response expectations with regard to the 45 most common side effects, including non**-**specific and specific complaints of endocrine therapy. Patients are instructed to indicate if and how strongly they expect to suffer from each potential side effect within the first three months of endocrine treatment.

*Self-efficacy expectations about coping* refer to the degree to which patients believe they are able to manage occurring side effects. They will be assessed using a modified version of the GASE**-**expect. Patients are asked to indicate their coping expectations for each of the 45 listed side effects (i.e., “Will I be able to manage occurring adverse symptoms?”).

*Treatment expectations:* Expectations about medicines in general as well as specific concerns and necessity beliefs about endocrine therapy will be assessed with the German version of the Beliefs About Medicines Questionnaire (BMQ) [19,50]. The BMQ has previously been used to assess medications beliefs in breast cancer patients [52]. Additionally, pre**-**treatment expectations regarding SEPT and ACG will be assessed using single items.

*Illness expectations:* Expectations e.g., about time course, consequences, personal and treatment controllability of breast cancer, will be measured with the brief illness perception questionnaire B**-**IPQ [53]. Each single item represents a scale (in addition to the above mentioned: concerns, emotional response, coherence and aspects of identity). The cause**-**scale was excluded. This questionnaire has previously been used in cancer patients [54]. As suggested, the words “illness” and “treatment” were replaced with “breast cancer” and “endocrine therapy” (Table 2).

### Additional variables

*Fear of progression* will be assessed with the short form of the of the Fear of Progression Questionnaire (PA**-**F**-**K) [55] consisting of 12 statements (e.g., being afraid of disease progression). It has shown good psychometric properties in breast cancer patients [56].

*Anxiety and depression* will be measured with the German Hospital Anxiety and Depression Scale (HADS**-**D) [38,39], which rates the severity of seven symptoms of anxiety and seven symptoms of depression over the past week and was designed for use in persons with physical illnesses. It has shown good psychometric properties in breast cancer patients [57].

*Treatment evaluation* will be measured in both intervention groups (ACG and SEPT). After every session, therapist and patient will rate their satisfaction with the unit using 12 items.

*Knowledge about the patient’s own tumor hormone-receptor status* will be assessed by one item [58].

*Somatosensory Amplification* is the tendency to perceive ambiguous sensory events as unpleasant and will be assessed with the Somatosensory Amplification Scale (SSAS) [59]. The scale consists of 10 items (e.g., “I am often aware of various things happening within my body”) and shows high validity and acceptable reliability in samples with breast cancer [60].

*Partnership quality* will be measured with one single item of the short form of the Partnership Questionnaire (PFB**-**K) [61] with reference to Terman [62] asking “How happy would you rate your partnership at the moment?”, which has been recommended for assessing satisfaction with a partnership.

### Data analysis

Missing values will be replaced using multivariate imputation techniques. A repeated measures multivariate analysis of variances (MANOVA) will be used to analyze treatment effects. To identify predictors of treatment outcome, multiple regression analyses will be conducted. To analyze pathways of the effects, mediator analyzes will be computed. Case studies will be performed to illustrate characteristics of the treatment processes from patients’ und therapists’ perspectives. Level of significance will be set at α = .05.

### Baseline demographic data

Preliminary baseline demographic data was assessed in N = 55 participating women with a primary breast cancer diagnosis. On average patients were 54.8 years old (SD=8.2, range=39-71 years), and mostly married or living with a partner (61.8%). Other patients were single (12.7%), widowed (5.5%) or divorced (20.0%). The majority of patients had primary education (63.6%), other patients finished secondary education (16.4%) or university education (20.0%).

More than half of the patients were diagnosed with stage I breast cancer (69.1%), further 27.2% of patients were diagnosed with stage II and additional 3.6% with stage III. Most patients (89.1%) received breast conserving therapy and only 10.9% mastectomy. A large group of patients (69.1%) was scheduled to undergo first-line endocrine treatment with tamoxifen (+/− GnRH-analoga). A third generation aromatase inhibitor (i.e. anastrozole, letrozole or exemestan) was recommended to the other patients (30.9%).

## Discussion

Although expectations have been found to predict the occurrence of side effects in cancer patients, this study presents the first randomized controlled trial evaluating a short**-**term cognitive**-**behavioral intervention to prevent side effects during adjuvant endocrine therapy by optimizing breast cancer patients’ expectations. The side**-**effect prevention training (SEPT) is compared with an attention control group (ACG) receiving supportive therapy and a standard medical care group (SMC) receiving standard treatment for breast cancer patients and additional oral and written information about adjuvant endocrine treatment. The primary outcomes are the occurrence of side effects three and six months after the start of intake of endocrine therapy. Further beneficial effects for quality of life, coping ability and adherence to medication are evaluated. Patients’ response expectations, expectations about coping ability and expectations about treatment and illness are analyzed as mediators. The study also gives some insights into characteristics of patients who benefit the most from SEPT and of patients at high risk of developing side effects.

If SEPT is found to be effective, it could be integrated into daily clinical practice. Effects of preventing non-specific symtoms and nocebo side effects may improve the quality of life during treatment, lead to better medication adherence, and thereby may help to reduce progression and mortality in breast cancer patients and decrease costs of treatment. The training could be delivered into health care settings and applied by trained and supervised health care professionals. In addition, the study will provide insights into pathways of clinical nocebo effects and non**-**specific side effects that may be applicable to other fields of illness and medication.

The study has some limitations that need consideration. First, our study design does not allow complete control regarding information patients may receive from their gynecologists about side effects of endocrine treatment, coping possibilities and about what happens while patients are in rehabilitation clinics. Another problem is that the study does not include patients who decide to start endocrine therapy before the baseline measurement. Presumably, patients in this group experience the strongest feelings of anxiety of progression and have more negative illness beliefs. This study does not provide conclusions about the efficacy of single treatment elements or differential indications. If treatment effects are robust, future studies are needed to analyze the particular influences of those factors.

The optimization of expectations might be a promising pathway to improve patients’ quality of life during medication intake. So far, this is the first study investigating a psychological prevention program for side effects with the explicit focus on patients’ expectations. The results will provide implications for a possible integration of evidence**-**based prevention training into clinical practice.

## Abbreviations

ACG: Attention control group; AET: Adjuvant endocrine therapy; ER/PR: Estrogen receptor/ progesterone receptor; BCT: Breast**-**conserving therapy; GnRH: Gonadotropin**-**releasing hormone; SEPT: Side**-**effect prevention training; SMC: Standard medical care.

## Competing interests

The authors declare that they have no conflict of interests that could have influenced the content of this report. WR received honoraria from Astra Zeneca, Heel, and Berlin Chemie for consultation and talks about placebo effects and medication adherence. YN received honoraria from Berlin Chemie and Atlantis Healthcare for consultation and talks about placebo effects and medication adherence.

## Authors’ contributions

PvB is a PhD student in the project, participated in writing the intervention manual, carries out the psychological interventions and drafted the manuscript. FS is also a PhD student, contributed to the intervention manual and carries out the psychological interventions. USA and WR are co**-**investigators and participated in the conception of the study. YN is the principal investigator, formulated the research questions, conceptualized the study design and wrote the intervention manual. All authors approved the final version of the manuscript.

## Pre-publication history

The pre-publication history for this paper can be accessed here:

http://www.biomedcentral.com/1471-2407/13/426/prepub
